# Investigating socially assistive systems from system design and evaluation: a systematic review

**DOI:** 10.1007/s10209-021-00852-w

**Published:** 2021-11-15

**Authors:** Shi Qiu, Pengcheng An, Kai Kang, Jun Hu, Ting Han, Matthias Rauterberg

**Affiliations:** 1 Department of Design, Shanghai Jiao Tong University, Shanghai, 800 Dongchuan RD. Minhang District China; 2 Department of Industrial Design, Eindhoven University of Technology, Eindhoven, Netherlands; 3 School of Design, Southern University of Science and Technology, Shenzhen, China; 4 School of Computer Science, University of Waterloo, Waterloo, ON Canada; 5 Department of Industrial Design, Nantong University, Nantong, China

**Keywords:** Assistive technology, Socially assistive system, Social interaction, Older adults, People with disabilities

## Abstract

*Purpose* The development of assistive technologies that support people in social interactions has attracted increased attention in HCI. This paper presents a systematic review of studies of Socially Assistive Systems targeted at older adults and people with disabilities. The purpose is threefold: (1) Characterizing related assistive systems with a special focus on the system design, primarily including HCI technologies used and user-involvement approach taken; (2) Examining their ways of system evaluation; (3) Reflecting on insights for future design research. *Methods* A systematic literature search was conducted using the keywords “social interactions” and “assistive technologies” within the following databases: Scopus, Web of Science, ACM, Science Direct, PubMed, and IEEE Xplore. *Results* Sixty-five papers met the inclusion criteria and were further analyzed. Our results showed that there were 11 types of HCI technologies that supported social interactions for target users. The most common was cognitive and meaning understanding technologies, often applied with wearable devices for compensating users’ sensory loss; 33.85% of studies involved end-users and stakeholders in the design phase; Four types of evaluation methods were identified. The majority of studies adopted laboratory experiments to measure user-system interaction and system validation. Proxy users were used in system evaluation, especially in initial experiments; 42.46% of evaluations were conducted in field settings, primarily including the participants’ own homes and institutions. *Conclusion* We contribute an overview of Socially Assistive Systems that support social interactions for older adults and people with disabilities, as well as illustrate emerging technologies and research opportunities for future work.

## Introduction

The domain of Human–Computer Interaction (HCI) has a long-standing history of developing assistive technologies for well-being [1]. According to the International Organization for Standardization (ISO) [2], assistive technology (AT) or assistive products include “devices, equipment, instruments and software especially produced or generally available, used by or for persons with disability.” In addition to persons with disability, the World Health Organisation (WHO) classifies five primary types of people who most need assistive technology [3]. These are (1) people with disabilities, (2) older adults, (3) people with noncommunicable diseases, (4) people with mental health conditions, and (5) people with gradual functional decline [3]. In our study, assistive technology addresses older adults and people with disabilities, aiming at maintaining or improving their functioning and independence, helping them achieve physical and mental health [3]. The developments in multisensory techniques, computer vision, and wearable technology have introduced various emerging assistive technologies to improve the life quality of older adults and people with disabilities. These assistive systems aim at fulfilling the essential needs of specific users in a broad scope of usage contexts and scenarios such as smart homes and healthcare systems for older adults [4], screen reader software and braille displays for blind people [5], assistive communication systems for children with autism spectrum disorder (ASD) [6], gesture-recognition systems for deaf people [7], as well as smart wheelchair systems for people with physical disabilities [8].

In many cases, studies involving assistive technologies focus on enabling fundamental capabilities or “survival” skills of specific user groups (e.g., facilitating visually impaired users to navigate a digital map ([9, 10]), or increasing the mobility of people with physical disabilities ([11, 12])). Relatively fewer studies ([13, 14]) have focused on the assistive technologies’ values of enhancing target users’ social interaction qualities. Nonetheless, neither people nor technology can exist in a social vacuum [15]. As social beings, people have an inherent desire to communicate and maintain social relationships with others [16]. Based on Abraham Maslow’s hierarchy [17], once people’s basic needs (i.e., survival needs) are satisfied, they will strive to satisfy higher-level needs, such as communication, love and sense of belonging in social circumstances. Insufficient social ties and communication can cause undesirable consequences, such as loneliness, depression, and social isolation in special-need users, such as older adults [18] and people with disabilities [19]. Thus, there is a substantial and rapidly growing demand for assistive technologies that can support social interactions.

An increasing number of existing examples have shown that assistive systems can satisfy users with special needs in social interactions. For instance, for older adults, there is a greater focus on healthy aging to help them maintain personal relationships and avoid experiencing feelings of loneliness [20]. As an example, a view-sharing system provides the continuous real-time changes of the shared outside views to promote social interactions between older adults living in a care home and people in local communities around [21]. An electronic picture frame could monitor older adults at home with unobtrusive sensors, and collect data about their health [22]. Through this way, older adults can maintain social connectedness with their caregivers. Other examples can be found regarding Socially Assistive Systems for people with disabilities, such as a haptic display that can convey facial movements to help blind people understand interaction partners’ facial expressions and emotions through vibration feedback [23]; a wearable device that allows blind people to perceive and react to gaze from conversation partners through haptic and visual feedback ([14, 24]); and a nonverbal communication application that supports real-time social distance regulation for children with ASD [25].

Most of the systematic reviews introduce assistive technologies focusing on solving special-need users’ basic needs in their daily living activities, such as dementia care [26], body rehabilitation [27], and navigation assistive technologies [28]. Only a few examples of the systematic reviews could be found to study Socially Assistive Systems, however, they all focused on a specific type of Socially Assistive Systems, and only for older adults. ([29–31]) reviewed Social Assistive Robots (SAR) that were designed for older adults. Other reviews summarized ICT technologies regarding how to reduce older adults’ social isolation and enhance their communication ([32, 33]). These reviews have been published recently, but do not take into account all different types of socially assistive technologies with diverse user groups. Socially Assistive Systems are helpful for all special-needs users, including older adults and people who have disabilities. Since research in this field is of growing interest, and every year a lot of new studies are published, there is a great need for writing a systematic review, classifying the existing studies, identifying the promising trends, and guiding the future design research. The generated knowledge will contribute to the HCI community.

In this paper, we present a systematic review to investigate Socially Assistive Systems from two primary aspects: system design and evaluation. In system design, we are interested in which kinds of assistive technologies have been used to address target users’ problems in social interactions. Since some social signals, such as gaze, facial expressions and vocal behaviors are not accessible to people with disabilities (e.g., blind or deaf people), we are particularly interested in how the assistive systems can help target users to perceive social signals. Moreover, we want to investigate how the Socially Assistive Systems are designed in terms of user-centered design and involvement of target users, since Sanoff (1990) claimed that any design aiming at improving the quality of users’ everyday life should consider participation through user involvement [34]. Aside from the descriptive aspects of system design, we are also interested in how the Socially Assistive Systems are evaluated. Our target users are faced with many challenges in the system evaluation stage. For example, older adults and people with certain disabilities are not easy to go to a specific location to attend a laboratory experiment. Different from other types of systems, Socially Assistive Systems often need to involve more than two people to investigate their social interactions, which increase the complexity and difficulty in system evaluations. Thus, it is valuable to know which evaluation methods have recently been used in Socially Assistive Systems and the applicability of each method.

To summarize, in this systematic review, we answer two primary research questions:

- ***RQ1***: Which kinds of assistive technologies are available to support target users’ social interactions, including perceiving social signals, and how to involve target users in their system design?
- ***RQ2***: Which types of the evaluation methods are used in Socially Assistive Systems and how to apply each method in system evaluation?

## Method

### Search strategy

A literature search was conducted in the following six databases: Scopus, Web of Science, ACM, Science Direct, PubMed, and IEEE Xplore. These databases were chosen because they provide full-text journals and conference proceedings of the most important conferences involving assistive technologies, social interactions, and their relations.

To seek out articles, we selected papers across two categories of Medical Subject Heading (MeSh) terms: “social interactions” and “assistive technologies”. According to MeSh terms, one of the synonyms of “social interactions” is “interpersonal relations”, which refers to “The reciprocal interaction of two or more persons”. Accordingly, several approaches can be used to allow or promote social interactions among people such as face-to-face conversations and exchanging messages though social media. In this review, we identified that only social interactions by means of assistive technology were considered. More specifically, it refers to any information systems that help people develop positive social interactions with each other [35]. Synonyms and spelling variations of “social interactions” and “assistive technologies” were used in several combinations and modified for the databases. Some search terms used in this search strategy were also derived from previous studies regarding social interactions [32] and assistive technologies ([26, 36]). Table 1 illustrates the example search strategy that was used for the ACM digital library. The searches were performed on article titles, keywords and abstracts. The search strategies for the other five databases resemble this. Relevant articles published in the past 22 years (January 2000–July 2021) were collected. Articles included refereed journal papers and peer reviewed articles that published in conference proceedings. Only English articles are included.

**Table 1:** Literature search strategy

Categories	Boolean search string
Social interactions	"social interactions" OR "social activity" OR "social connectedness" OR "social connectivity" OR "social isolation" OR socially OR "interpersonal relation"
	AND
Assistive technologies	"assistive technology*" OR "assistive device*" OR "assistive product" OR "assistive application" OR "technical aid" OR "assisted living" OR "self-help device"

### Article selection

The selection process was conducted according to the guidelines of The Preferred Reporting Items for Systematic Reviews and Meta-Analyses (PRISMA) Statement [37]: (1) a computerized search strategy (Table 1) was performed from October 2019 to July 2021; (2) SQ removed duplicates, screened titles and abstracts of the remaining articles; (3) Two independent coders (i.e., SQ and PCA) screened, analyzed and evaluated the full-text articles. After that, they should reach a consensus to decide which articles fit the inclusion criteria. Discrepancies were resolved by discussions between the two coders.

The articles that met at least one of the following inclusion criteria were included:

- Articles presented system design of Socially Assistive Systems;
- Articles included system evaluation of Socially Assistive Systems.

The articles that met at least one of the following exclusion criteria were excluded:

- Reviews and books;
- Theoretical articles;
- Concept articles;
- Market surveys;
- No information systems;
- Information assistive systems, but not for social interactions;
- Information systems are not designed for older adults and people with disabilities;
- Articles are not written in English;
- Articles are less than four pages;
- Duplicate reports of the same study in different sources;
- Robotic systems.

Among different kinds of Socially Assistive Systems, robotic systems include a large number of studies, such as Socially Assistive Robots for older adults ([29, 30, 38]) and children [39]. Some existed review papers have reported such field, so we did not include robotic systems in our systematic review.

### Data extraction

After discussions of the authors, a template was determined for data extraction. Based on the research questions, we extracted data from two primary aspects: system design and evaluation. In more detail, the template included the following categories (see Appendix for summary of paper lists and features):

- **HCI technologies** include wearable technologies, multimedia technologies, and other HCI technologies that support social interactions, such as virtual reality (VR) and augmented reality (AR) technologies;
- **Social signal perception** refers to the system design that help target users to perceive nonverbal signals, such as facial expressions and head pose;
- **User involvement** refers to whether and how the users and stakeholders involve in the design procedure, such as target users, caregivers, experts and families;
- **Evaluation** consists of evaluation type and time span. Four evaluation types are identified: (1) *laboratory experiment*, (2) *field experiment*, (3) *qualitative study in artificial setting*, and (4) *qualitative study in field setting*. Time span includes hours/days/weeks/months.

## Results

### Overview

**Fig. 1:**
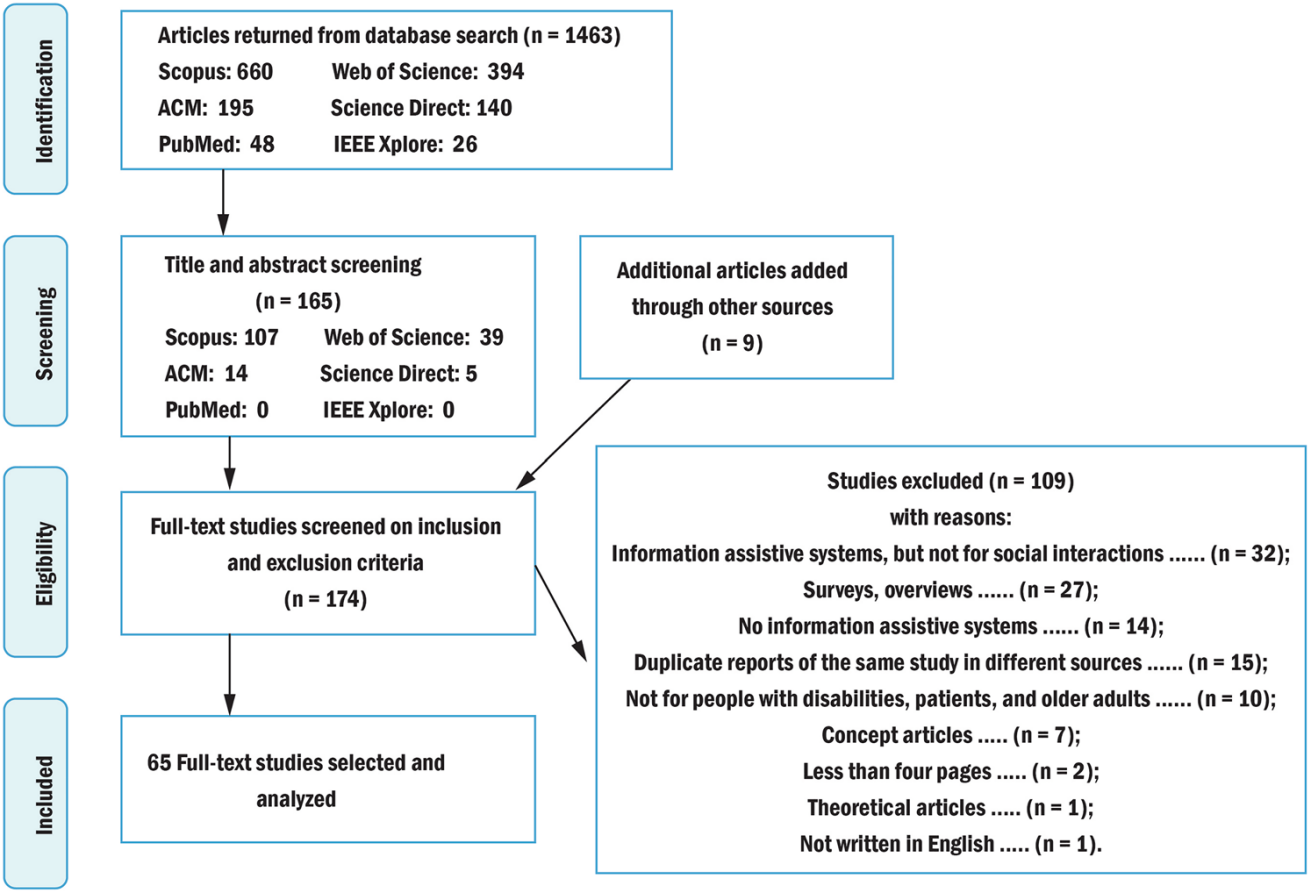
PRISMA [37] flowchart of the results of the literature search

Figure 1 shows an overview of the results during the different stages of selecting articles. Initially, 1463 articles were identified according to the search strategies. After title and abstract screening and removing duplicates, 165 articles remained. Next, we selected full-text articles according to the inclusion and exclusion criteria. In total, 56 articles were included, which directly related to Socially Assistive Systems. We manually searched the references for included articles, and nine articles were selected after hand searching of references. Finally, 65 articles were considered for the systematic review. The primary features of each study are summarized in the Appendix.

### System design

#### HCI technologies

**Fig. 2:**
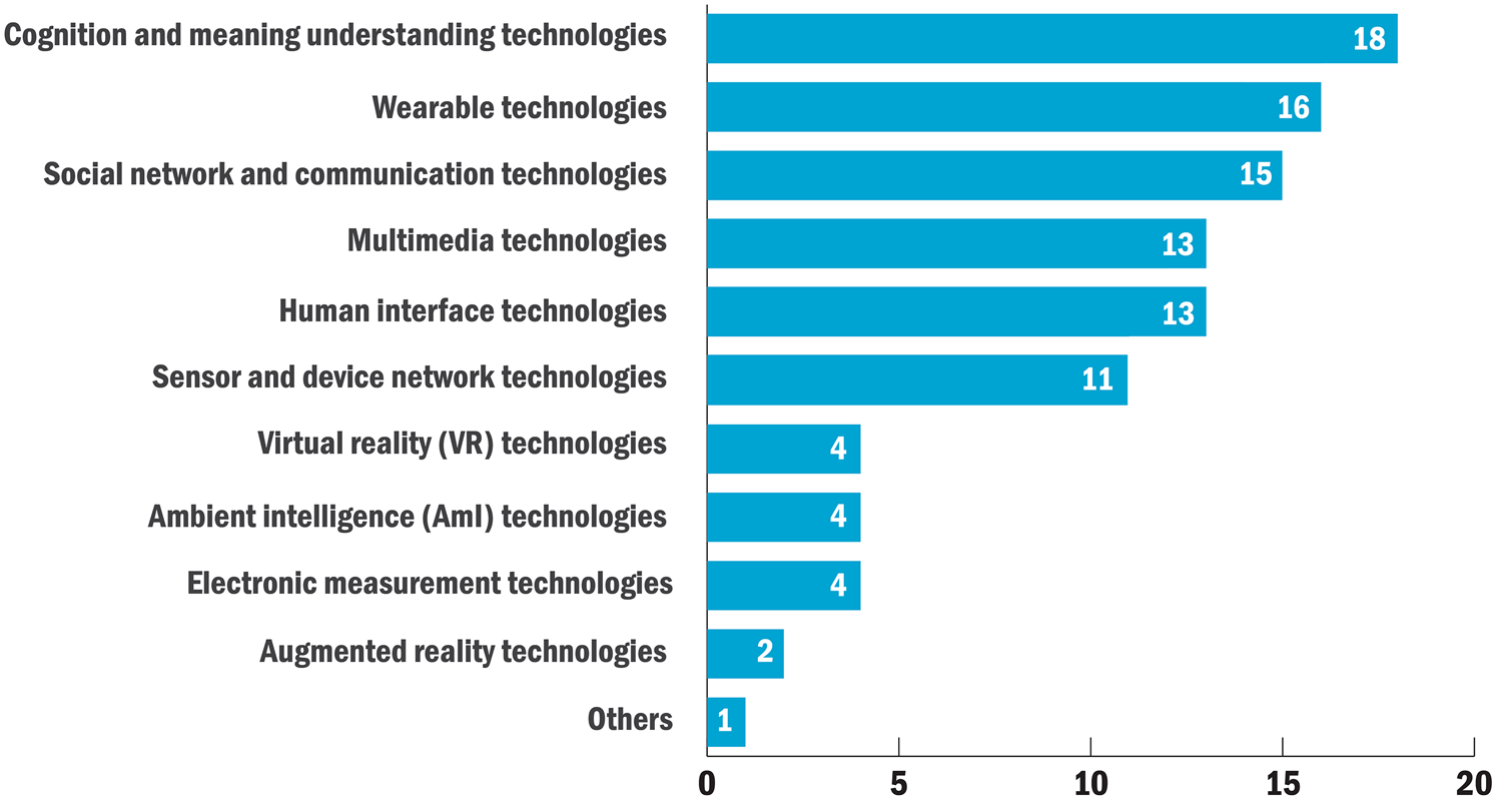
Types of HCI technologies

**Fig. 3:**
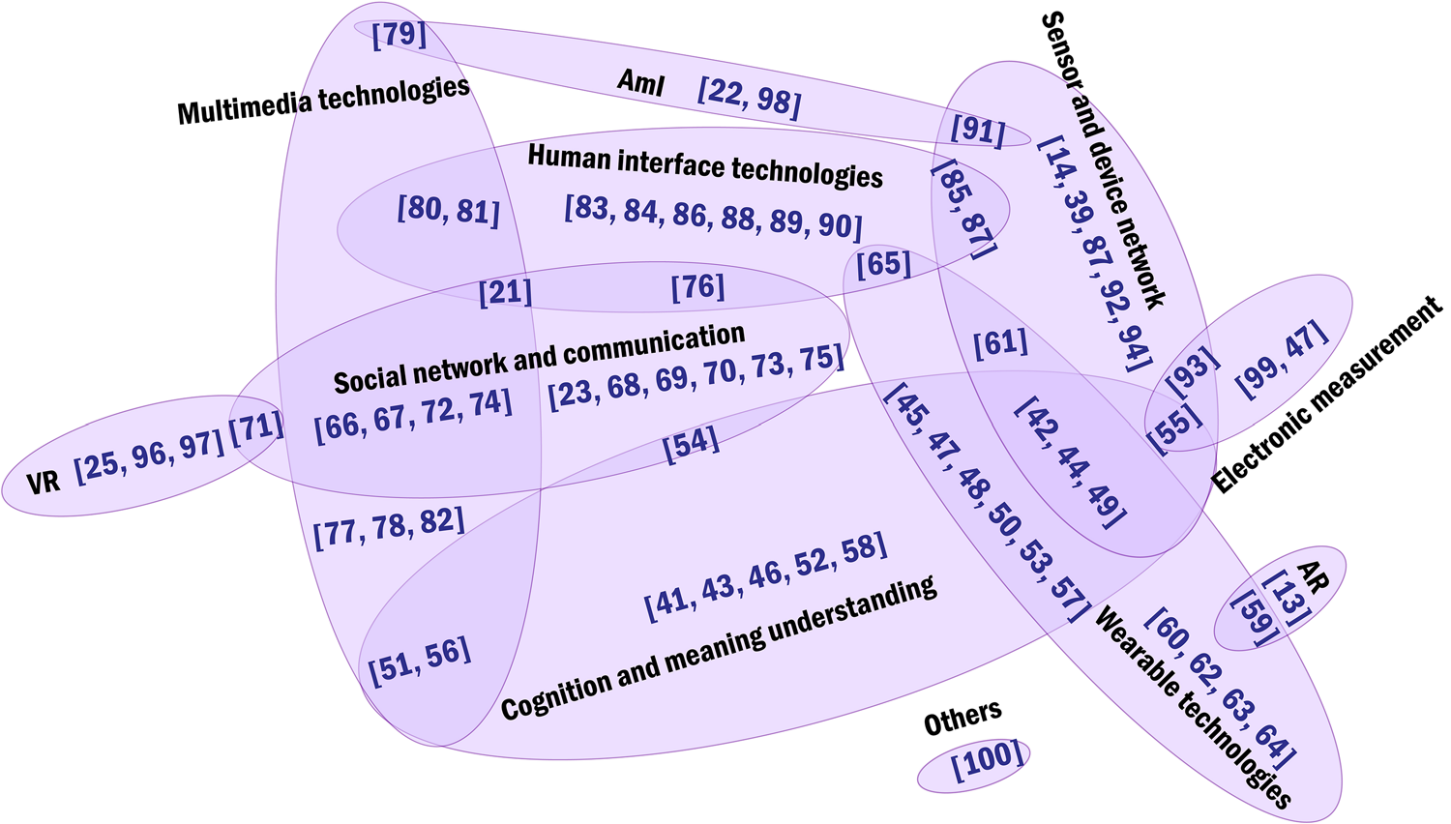
A Venn diagram illustrates the relationships of multiple HCI technologies extracted from reviewed articles

Since a great proportion of Socially Assistive Systems adopted Information and Communication Technology (ICT) for its capacity to socially connect people, the main categories of the HCI technologies used in the reviewed studies are classified based on a taxonomy of ICT proposed by Inaba and Squicciarini [40]. Seven out of 13 technology areas in the original taxonomy were removed, mainly because they are underlying technologies that are not very relevant to human interaction (e.g. Large-capacity and high-speed storage). Besides, we add five kinds of emerging technologies in the HCI community because they are not included in this taxonomy but repeatedly mentioned in the reviewed articles. Finally, the established classification includes 11 technologies (Fig. 2): (1) *social network and communication*, (2) *sensor and device network*, (3) *cognition and meaning understanding*, (4) *human interface*, (5) *electronic measurement*, (6) *wearable technologies*, (7) *multimedia technologies*, (8) *Virtual Reality (VR)*, (9) *Ambient Intelligence (AmI)*, (10) *Augmented Reality (AR)* and (11) others. Table 2 shows the reviewed studies that applied each type of technology. Since some proposed solutions are the synthesis of multiple technologies, we mainly assign the studies to the technology areas specifically mentioned by the researchers. Usually, less than three core technologies were extracted from each study. A Venn diagram uses the circles to show the relationships among multiple HCI technologies among reviewed articles (Fig. 3).

**Table 2:** Classification of HCI technologies

Technology areas	Encompass	References
Cognition and meaning understanding	Facial/gesture tracking & recognition	[41–53]
	Text & code recognition	[54, 55]
	Voice recognition	[56]
	Identity recognition	[57]
	Image description	[58]
Wearable technologies	Head-mounted devices	[42, 45, 47–50, 53, 59–61]
	Smart belts	[44, 45, 49, 50, 60]
	Smart watches/bands	[57, 62, 63]
	Smart jewels	[64]
	Smart vest	[65]
Social network & communication	Online social platforms	[21, 66–72]
	Video/audio communication	[66–69, 73–76]
	Messaging communication	[23, 54, 66, 68, 69, 75]
Multimedia technologies	Media playing/recording/sharing system	[21, 51, 55, 66, 67, 72, 74, 77–81]
	Interpretation systems	[56, 82]
Human-interface technology	Haptic interface	[65, 83–85]
	Tangible interface	[21, 76, 86–88]
	Touchscreen interface	[80, 81, 89]
	Semi-transparent Video Interface	[90]
Sensor and device network	Distributed/Multiagent systems	[42, 87, 91–94]
	Multi-device systems	[14, 39, 44, 49, 61, 85]
Virtual Reality (VR)	Therapeutic VR applications	[25, 71, 96]
	360 videos and images	[97]
	Immersive touring application	[97]
Ambient intelligence (AmI)	Ambient displays	[22, 79, 91]
	Ambient lightings	[22, 98]
Electronic measurement	Social environment tracking and navigation	[55, 93, 99]
	Head gesture recognition	[47]
Augmented Reality (AR)	Mobile application	[13]
	Technology mediated sight	[59]
Others	Affective avatar taxonomy	[100]

*Cognition and meaning understanding technologies* were mentioned in 18 studies. It is a subset of the broader field of artificial intelligence that simulates the functions of the human brain, including computer vision technologies, natural language processing and pattern recognition [40]. Computer vision technologies represented the largest proportion in this area, and most target users were people with visual impairments. They were mainly used to capture, track and recognize non-verbal social cues such as eye contact, facial expressions and head gestures ([41, 42, 44–50, 52, 53]). One study also applied computer vision to assist people with memory loss to identify social partners [57]. Pattern recognition technologies were applied to support blind users on social networks by recognizing digital images and generating descriptions [58]. Printed patterns such as text and codes could also be recognized for further social contacts, which could be used by older adults [54]. In addition, voice recognition is a very useful technology for deaf people, but only one study explored in this area [56].

*Wearable technologies* represented the third largest proportion (16 studies). They were often applied with computer vision technologies to capture visual cues and provide haptic or audio feedback. A typical form is using smart glasses alone ([14, 42, 47, 53, 59, 61]) or with smart belts ([45, 49, 50]). Smart bands and watches are also very common solutions ([57, 62, 63]). Apart from collecting user data, they were mainly used as a personal reminder to improve social skills for the people with memory loss or children with ASD. A smart vest was proposed in [65] to provide tactile feedback for the user with deaf-blindness. Only one study addressed the esthetic values and socio-cultural aspects of wearable technologies [64].

*Social network and communication technologies* were used in 15 studies. They were mainly embedded in software applications to assist tele-communications, which is effective to reduce distance barrier and social isolation. Therefore, the great majority of the target users in this category were older adults and people who had motion disabilities. Typical communication technologies were applied to establish direct social contact including video/audio chatting ([66–69, 73–76]) and messaging ([23, 54, 66, 68, 69, 75]). Eight studies developed online platforms where people could create their own profiles, build relationships with other users, and engage in social activities together ([21, 66–71]). In [21] a case is also mentioned aiming to enhance the social connectedness of nursing home residents by transferring real-time photos of outdoor sceneries.

*Multimedia technologies* were applied in 13 studies. They mainly refer to the technologies that use text, graphic, animation, and sound to deliver information. Seven studies ([21, 72, 74, 78–81]) presented media playing systems to provide meaningful content for older adults as social stimulus. Multimedia technologies were also frequently used to record digital content, which could be shared by older adults or people with language impairments to enhance their social connectedness through self-disclosure ([55, 67]). The recorded media content could also be used to improve the social skills of people with ASD ([51, 77]). Two studies developed multimedia applications to interpret sign languages for deaf people ([56, 82]).

*Human interface technologies* were specially mentioned in 13 studies. Haptic interfaces were explored in four studies mainly for people with visual impairments. They presented a mapping between visual cues and vibrotactile representations with devices such as smart belts and ergonomic mesh chairs ([65, 83–85]). Four studies integrated social application with tangible interfaces to increase the attractiveness and reduced the technological barriers for older adults and children ([21, 76, 86, 87]). Multi-touch interfaces were increasingly used to encourage co-located collaboration. They were used in three studies to support relationships between people with dementia and caregivers or enhance the social skills of children with ASD ([80, 81, 89]). One study developed two applications with semi-transparent video interfaces to assist deaf people in local group meetings and remote personal meetings [90].

*Sensor and device network technologies* were applied in 11 studies. Apart from the multi-device systems that were used with wearable technologies ([14, 39, 44, 61]), a large proportion of the solutions were distributed or multi-agent systems that were developed mainly based on a client/server architecture. The client devices could interact with each other online or offline, and their status could also reflect on the server device. They were usually designed for communities such as care homes or classrooms to enhance social awareness of older adults or children with ASD ([87, 91, 92]).

*Virtual reality (VR) technologies* were adopted in four studies. Two studies developed immersive therapeutic VR systems for children with ASD ([25, 96]), and one study designed an online VR platform for people living with spinal cord injury [71]. In addition, one study reported the positive effect of VR applications on older adults suffering from social isolation [97].

*Ambient intelligence (AmI) technologies* were used in four studies. As a key technology in the ambient assisted living environment, they were mainly applied to enhance the social awareness and interconnectedness between older adults who live alone and their friends, family members or caregivers with ambient displays or ambient lightings ([22, 79, 91, 98]).

*Electronic measurement technologies* were adopted in four studies. They refer to the technologies that collect, process and analyze electronic signals such as radio waves, Wi-Fi signals and ultrasonic waves. The applications were designed for people with physical disabilities or visual impairments. They were primarily implemented in wearable devices or mobility aids to measure the presence, location, distance or velocity of users’ social partners ([55, 93, 99]). One study presented two new methods for real-time sonification of head movements and head gestures [47].

*Augmented reality (AR) technologies* were applied in two studies. One study developed a mobile AR game application for children with ASD to improve their social skills [13]. The other study proposed technology mediated sight to highlight meaningful daily information for people with visual impairments [59].

Only one study did not directly involve any type of technology mentioned above. It proposed a human-avatar taxonomy to support social communication disorder evaluation [100].

#### Social signals perception

**Fig. 4:**
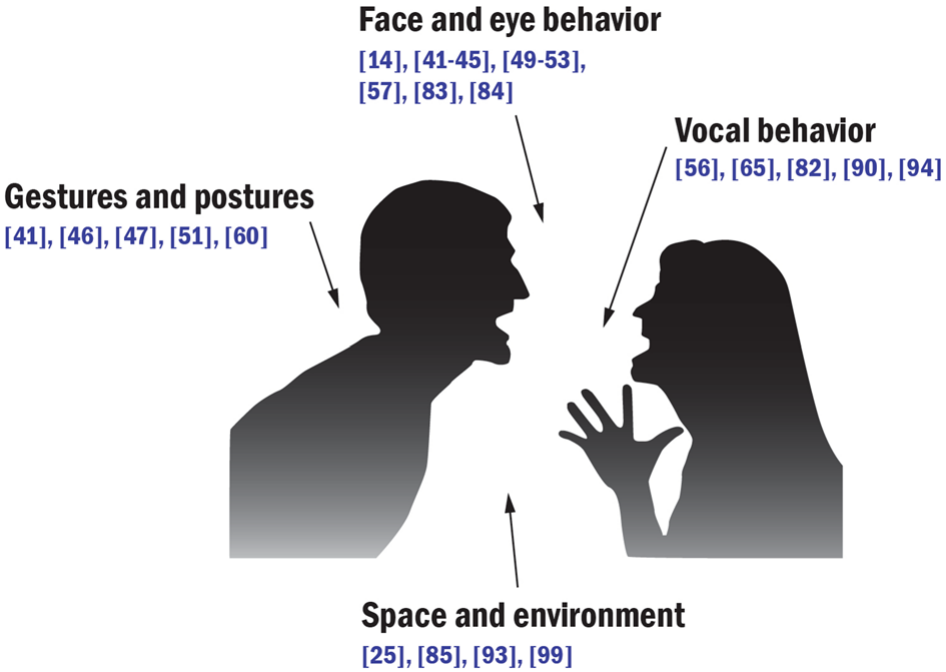
Socially Assistive Systems for perceiving different kinds of social signals

Social signals are defined as “the expression of ones attitude towards social situation and interplay” [101], which are shown through a variety of verbal and nonverbal cues. Vinciarelli et al. [101] presented the taxonomy of social signals, including (1) *face and eye behavior*, (2) *gestures and postures*, (3) *vocal behavior*, (4) *space and environment*, as well as (5) *physical appearance*. According to the taxonomy of social signals, we classify the Socially Assistive Systems which are used to support social signals perception. In our review, 27 out of 65 studies (41.54%) presented Socially Assistive Systems to perceive (1) *face and eye behavior* (14 studies), (2) *gesture and postures* (five studies), (3) *vocal behavior* (five studies), as well as (4) *space and environment* (four studies) (Fig. 4).

*Face and eye behavior* A total of 14 studies ([14, 41–45, 49–53, 57, 83, 84]) explored Socially Assistive Systems to perceive face and eye behavior of interaction partners. Among them, the majority of these systems supported identifying face behavior. Seven studies addressed people with visual impairments to perceive typical facial expressions ([41–45, 53, 84]), facial movements [83], and head pose [53] of their conversation partners or pedestrians. In [51], a sharing system was targeted at children with ASD, to help them recognize facial expressions; [57] presented a mobile phone-based app to identify people’s face, assisting people with dementia to remember the names and relationships of their interaction partners. Three studies ([14, 49, 50]) were regarding Socially Assistive Systems for gaze perception; [49] used a wearable device to help a blind person perceive eye gaze from a sighted person in dyadic conversations. The prototype of [14] simulated eye gaze of blind people, allowing them to establish “eye contact” with sighted conversation partners. In [50], researchers developed a multimodal assistive system to inform a blind person in real-time whenever someone was looking at her.

*Gesture and postures* Five studies presented systems for gesture and postures perception. Head pose estimation technology was used to help people with visual impairments ([41, 46, 47, 60]) and children with ASD [51] to perceive typical head gestures/movements, such as nodding and shaking. For example, [60] developed a Social-Aware Assistant (SAA) to convey the head nodding of conversation partners by using a vibratory belt, to enhance face-to-face interaction between blind and sighted people.

*Vocal behavior* Five studies ([56, 65, 82, 90, 94]) presented systems that could support deaf people to perceive vocal behaviors. These systems could transfer the voice to sign language ([56, 90, 94]), or sign language to the voice [82], so as to establish communication between deaf and hearing people. In [65], researchers developed a Tactile Board focusing on translating the voice into vibrotactile signs, in order to facilitate communication for deafblind people.

*Space and environment* Four studies ([25, 85, 93, 99]) described the systems for social distance perception. In ([85, 93]), researchers developed Socially Assistive Systems for blind people, to help them identify the distance of interaction partners and initiate social contact; [25] was regarding providing a real-time support for the proximity regulation of children with ASD, to adjust their social behavior to be more in line with local cultural norms. In [99], a social following control system supported a good conversation distance between the wheelchair user with physical disability and a companying person.

#### User involvement

*Involvement of end-users and stakeholders* While it is conventional for researchers and developers to validate their designed assistive technologies with end-users, it is not a common case for related research to feature end-user involvement in the design phase. In our review, 22 out of 65 (33.85%) studies reported end-user involvement in the design stage. Among these studies, nine (40.91%) also reported the involvement of stakeholders in addition to the end-user group. These stakeholders included professionals who provided care or services to the target groups, such as caregivers (e.g., [21, 55]), educators (e.g., [89]), therapist (e.g., [92]), etc. On the other hand, these stakeholders also included domain experts who had specialized in certain aspects of the target design challenge, such as psychologist, technologist, designers, ethicist (e.g., see [64]). For instance, while targeting the special learners as their end user group, [92] also involved the educators and therapists in their participatory design activities; [73] conducted expert interviews to integrate domain experts’ perspectives in design. Similarly, [25] included two caring staff members in their participatory prototyping with 10 developmentally disabled adults.

The benefits of involving stakeholders in addition to the end users can be generalized two-fold. First, these stakeholders possess rich practical experiences or scientific understandings about the design context, which could compensate the knowledge and expertise of the developers. Second, for some targeted end-users, such as people with dementia, or children with ASD, it can be difficult for them to fully express their tacit needs and experiences. With the stakeholders’ help, the development team could better discern the target group’s needs and experiences.

*User-centered approach and participatory design* In general, two types of end-user involvement can be recognized from the reviewed papers: 16 out of 22 studies (72.73%) described a user-centered approach, and six out of 22 studies (27.27%) highlighted a participatory design (or codesign) approach. The major difference between the two concerns the role of the end-users. In a user-centered approach, end-users are involved as the providers of the contextual information, which helps the designers to better understand the design challenge and requirements. Whereas in a participatory design approach, in addition to providing contextual information, end-users also actively share the role of designers to formulate the design solution together. In user-centered development, various techniques have been used to understand the needs, attitudes, or experiences of the end users, including interviews (e.g., [65, 75]), ethnographic studies (e.g., [55]), focus group (e.g., [22, 80]), trial and customization (e.g., [88]), feedback sessions (e.g., [90]), etc.. Whereas in a participatory design approach, besides the above mentioned techniques to gather user-centered insights, the end-users are also invited to participatory, or co-design workshops, to generate design concepts or solutions together with a group of stakeholders (e.g., see [73, 91, 92]). The outcome of the participatory design workshops could take the form of some low-threshold design artefacts (like sketches, drawings, or collages), as well as mockups (e.g., see [91]), or low-fidelity prototypes (e.g., see [25]).

*End-user involvement in different stages of design* The reported user and stakeholder involvements also took place in different stages along the design process, from rather early moments of ideation all the way to the later stages which overlapped with design validation, For example, [94] utilized a focus group in an early stage to identify difficulties of deaf people in using assistive technologies. Whereas, addressing a similar context, [90] involved deaf participants in a session after the ideation to gather their feedback on the design concept. Moreover, several studies also featured end user and stakeholder involvements across multiple stages along their design process. For example, in [25] (targeted at developmentally disabled adults), the researchers first performed card sorting with design partners to gather user requirements. Subsequently, role play sessions were conducted with two participants to build a lo-fi prototype. Finally, the hi-fi prototype was built together with target users and their caring staff members.

*Engagement with real-world contexts* In addition to including end users and stakeholders in design, seven out of 22 papers (31.82%) also highlighted their in-depth engagement with the real-world contexts of their target group. For example, as reported in [55], a two-week design ethnography study was conducted, in which the researchers shadowed three participating children to gather field insights into their daily life. Similarly, in [92], the researchers observed nine classrooms to gain on-the-ground knowledge about the special education context. For another example, in [68], the researchers conducted an observational study to closely understand how a game is learnt and played by older adults in a senior center.

#### Summary for RQ1

In response to RQ1, we summarize the findings related to system design in the following.

Eleven types of HCI technologies were identified that supported social interactions for target users. The top three frequently used HCI technologies in Socially Assistive Systems were (1) cognitive and meaning understanding technologies (17.82%), (2) wearable technologies (15.84%), as well as (3) social network and communication technologies (14.85%). Among them, the most common was cognitive and meaning understanding technologies, which were often applied with wearable devices for compensating vision, hearing, and memory loss. Among different types of social signals, the majority of Socially Assistive Systems were designed for target users to perceive face and eye behaviors of their interaction partners. Nearly one-third of the analyzed studies involved end-users and stakeholders in the system design stage. Among reviewed studies, there were two types of end-user involvement: (1) user-centered approach and (2) participatory design.

### System evaluation

**Fig. 5:**
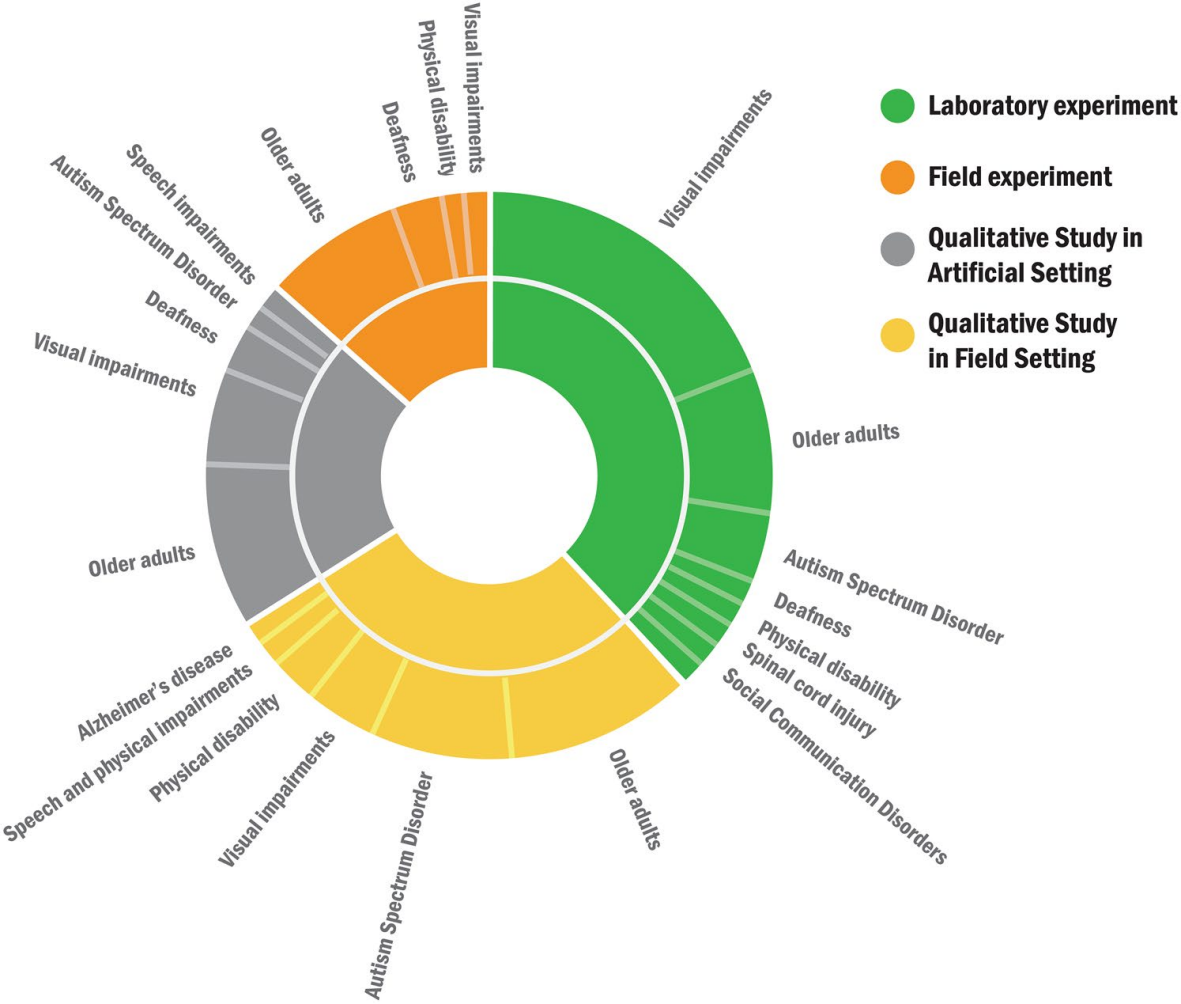
Evaluation methods for target users

We categorize system evaluations regarding Socially Assistive Systems based on (1) whether they were experimental studies or qualitative studies and (2) whether they were conducted in the lab or in the field. Thus, evaluation methods for Socially Assistive Systems are classified into four categories (Table 3): (1) *Laboratory experiment* (27 studies), (2) *field experiment* (10 studies), (3) *qualitative study in artificial setting* (15 studies), and (4) *qualitative study in field setting* (21 studies). Figure 5 shows the pie chat of four evaluation methods used for target users.

**Table 3:** Categories of evaluation methods

System evaluation		Reference
Laboratory experiment (27)	User system interaction (18)	[14, 25, 41, 42, 49, 57, 60, 70, 71, 73, 79, 81, 84, 87, 94, 96, 98, 100]
	System validation (9)	[43]–[47, 83, 85, 93, 99]
Field experiment (10)		[52, 53, 56, 66, 69, 74, 75, 78, 82, 97]
Qualitative study in artificial setting (15)		[22, 50, 54, 57–59, 64, 67, 68, 72, 77, 78, 80, 90, 95]
Qualitative study in field setting (21)		[13, 21–23, 51, 55, 58, 61–63, 66, 75–78, 86, 88, 89, 91, 92, 95]

#### Laboratory experiment

This category consists of two sub-categories: (1) *laboratory experiment for user system interaction* (18 studies), and (2) *laboratory experiment for system validation* (9 studies).

1. Laboratory experiment for user system interaction.
  In this category, user experiments were conducted under highly controlled condition, focusing on how to evaluate perceptions or experience of users towards Socially Assistive Systems. A total of 18 studies were targeted at people with visual impairments ([14, 41, 42, 49, 60, 84]), older adults ([70, 73, 79, 81, 98]), and children with ASD ([25, 87, 96]). Other four studies were regarding people with memory loss [57], spinal cord injury [71], Social Communication Disorders (SCD) [100], and deafness [94].
  In most situations, researchers involved representative users that directly reported their user experience and perceptions in laboratory experiments ([41, 60, 70, 71, 73, 79, 81, 84, 87, 94, 96]). For example, [73] investigated how to enhance intergenerational interactions between older adults and their grandchildren while online platform. Grandparents directly reported their user experiences with the platform, such as engagement, social connectedness and social presence. In certain cases, representative users were difficult to study and report directly about their own behaviors, attitudes and intentions, so proxy users provide a feasible solution for running such user experiments ([14, 25, 42, 49, 98, 100]). In [49], researchers tested blindfolded participants rather than real blind participants in their initial study. In [25], researchers evaluated whether immersive VR applications could provide effective real-time support for improving social distance for children with ASD. In this case, their mothers were treated as proxy users, to report their children’s user experience of using immersive VR applications.
  In this category, only three studies clearly mention that they received an approval from the ethics committee ([57, 60, 84]); [57] was a clinical trial to investigate people with memory loss. Both participants and their caregivers were recruited from the university, and the Institutional Review Board (IRB) of the university approved this study. In [84], researchers recruited a total of eight blind participants to attend the study. The research protocol was approved by an IRB. Similarly, in [60], researchers followed ethical standards provided by the American Psychological Association (APA). The ethics committee of the university approved the research protocol. Most studies (*N* = 15; 88.24%) used the consent forms for older adults and people with disabilities. The written consent was not always suitable. As reported in [81], the participants with dementia were first asked to provide a written consent, and if not feasible, verbal consent was obtained and witnessed by a neutral third party. Besides, in [70], all elderly participants were required to provide an approval by their family doctor to allow them to attend the study.
2. Laboratory experiment for system validation
  In this category, studies focused on evaluating system validation. Nine studies were targeted at people with visual impairments ([44–47, 83, 85, 93]), physical disability [99], and low vision, Alzheimer’s disease, and autism spectrum disorder [43].
  The majority of studies ([43–47, 83, 85, 93, 99]) tested the recognition rate of the target system. For example, in [83], researchers conducted a single trial to test the system accuracy of identifying facial features. Additionally, [99] investigated how to maintain a good conversation distance between the wheelchair and the companying person. Researchers tested the wheelchair control system to examine the feasibility of the pose detection algorithm.
  Among nine studies, six of them used proxy users in the preliminary experiment ([46, 47, 83, 85, 93, 99]). Generally, researchers tested system function with non-disabled participants instead of representative participants who had disabilities, such as people with visual impairments or physical disability. None of nine studies clearly reported an ethical protocol.

#### Field experiment

According to [102], field experiment refers to studies using an experimental design in a real-life environment. The experimenter still needs to manipulate at least one independent variable. A total of 10 studies used field experiments to evaluate Socially Assistive Systems, which were targeted at older adults ([66, 69, 74, 75, 78, 97]), people with deafness ([56, 82]), physical disabilities [52], and visual impairments [53].

These studies were conducted in various field settings. The most common was the participants’ own homes and their living communities ([66, 69, 74, 75, 82, 97]). Other places included institutions ([52, 75, 78]) and realistic situations for testing purposes ([53, 56]). For example, in [66], researchers evaluated 19 older adults in homes to use a communication system prototype over 10 weeks. Similarly, researchers visited older adults in their own homes to investigate Uniper-Care Technology (UCT) for enhancing social connectivity and entertainment. Most of these field experiments (*N* = 8; 88.89%) lasted for a long period of time, from several weeks ([56, 66, 69, 74, 75, 97]) to several months [82].

Two out of 10 studies clearly report that they were approved from the ethical committee ([52, 69]). For example, [69] recruited elderly participants through elderly associations and retirement homes. The study was approved by the ethical committee at each of the researchers’ universities. Other studies ([53, 66, 74, 75, 78, 82, 97]) mentioned that they used an consent form in their experiment. For example, in [78], a responsible relative signed the consent form to agree the participants with dementia to attend the study. Additionally, the nursing home manager and the activity facilitators consented to their participation in the study.

#### Qualitative study in artificial setting

The category of qualitative study in artificial setting is referred to studies which conducted qualitative evaluation of the design outcome (e.g., concepts or prototype systems) in a setting that was not the naturalistic environment lived in by the end-users. This category was targeted at various end-user groups, including older adults with needs for socializing activities ([22, 54, 68, 72]) or living with dementia ([57, 78, 80]), children with ASD [77], as well as people with visual impairments ([50, 58, 59, 95]), hearing loss ([64, 90]), or speech impairments [67].

Multiple types of such artificial settings could be seen in this category. First, five studies created ad-hoc settings for a group session: such as focus groups, or participatory design workshops ([50, 64, 72, 80, 90]). They often aimed for eliciting thick discourses and in-depth reflections from the target group (and their related stakeholders). For example, [64] introduced an evaluation approach which combined iterative participatory design workshops and a public exhibition, involving both the target group (people with hearing impairments) and domain experts. This subset of studies also featured a relatively close participation of the end-users and related stakeholders in the process: that was, participants were not only asked to evaluate a developed system, but also invited to participate in the iteration or modification of the evaluated design. For instance, in [80], focus groups were organized for the target group (people with dementia) and their caregivers to assess the designed interface and select relevant design elements.

The second subset in this category concerns four studies introducing a new form of social event or activity (e.g., a game) for end-users, which was outside their daily routines ([68, 77, 78, 95]). For example, [95] developed an audio-based augmentation system to enhance both blind and sighted players’ experiences in badminton. The study organized several game sessions for the target group to gather their experiences of the system. Similarly, [68] introduced a new social game to older adults to study their behaviors in, and responses to this newly learnt activity.

The last subset in this category is associated with five studies that adopted a simulation, or in-lab setting to qualitatively evaluate the developed systems with users via methods such as interviews ([22, 54, 57, 58, 67]). As an example, [22] constructed a simulation environment of home to invite the target group (older adults) to experience the developed system which was aimed for enhancing their social connectedness. For another example, an AI application developed for people with visual impairments to interact with social network photos was evaluated in [54] through interviews conducted in an in-lab environment. Among all studies from this category, only a few (five out of 15) provide information in regard to the ethical conduct with the target group. Namely, all three studies involving older adults with dementia briefly described the acquisition of participation consent (from responsible relatives [78] or caregivers and end-users [57]), or ethic approval from relevant authority [80]. One study supporting socialization of older adults described the reception of end-user consents [72]. One study targeted at people with hearing impairments mentioned the involvement of an ethicist in workshops [64].

#### Qualitative study in field setting

There are 21 studies in total in this category. We found the great majority of these studies were conducted out of age-related concerns. Sixteen studies were targeted at older people ([21, 22, 61, 66, 75, 76, 78, 86, 91]) and children ([13, 51, 55, 62, 77, 89, 92]). Two studies aimed at designing communication systems for the people with disabilities, and they still recruited teenagers as their participants ([23, 88]). Only three studies did not specially mention the age of their target users, which focused on people with vision impairments ([58, 63, 95]).

Most studies were carried out in institutions, such as care centers ([21, 61, 62, 75, 78, 86, 91]) and schools ([13, 23, 55, 77, 89, 92]). Three studies on people with dementia ([61, 78, 86]) and one study on children with ASD [62] mentioned that their participants were directly recruited by institutions according to research needs. The participants of two studies were recommended by institutions and selected by researchers ([55, 75]). Two studies recruited participants through independent research or social organizations ([66, 88]). In addition, our review shows that the evaluation with older adults were usually in the form of group activities organized and facilitated by caregivers ([76, 78, 86, 91]), while other studies in real-world setting were conducted with much less external assistance. It might because older people have more difficulties in accepting and using new technologies than the younger generations.

In spite of the advantages of field setting, researchers often have ethical challenges to collect data in participants’ daily life. To address this, signing consent is a common way to inform the participants of ethical issues ([63, 66, 88]). Besides, many researchers drafted research protocols that needed to be approved by related institutions or stakeholders before field trials ([13, 62, 63, 75, 88]). To protect the participants’ privacy in using communication applications, their real identities and content of conversations was only accessible to them and their social partners ([22, 66, 75, 89]). Given the specialty of children with ASD, one study mentioned that the participants were not informed of the purpose of the study to avoid stressing them [62]. Another study confined the enrolled students in a separated area to avoid disturbing the non-participants [13].

**Fig. 6:**
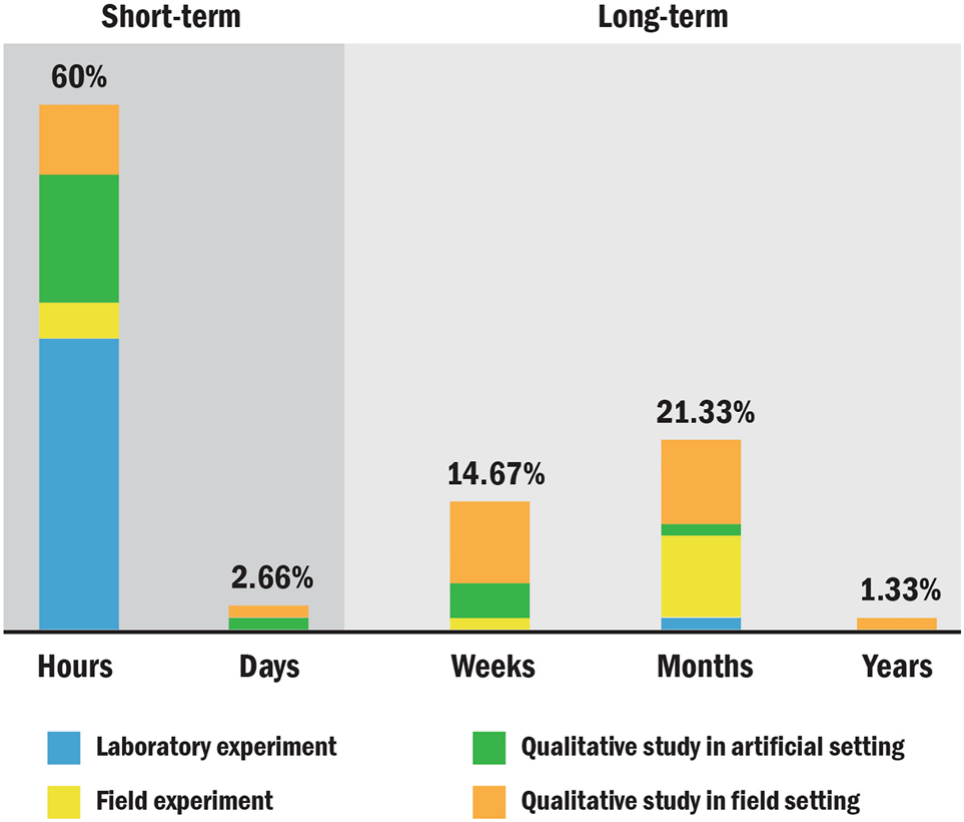
Time span of each evaluation method

Overall, the time span of evaluating Socially Assistive Systems ranged from several hours to over one year (Fig. 6). Most studies (*N* = 45; 60%) report several hours, while 16 studies report several months and 11 studies took several weeks. Two studies took several days and one study lasted for over one year. Short-term studies (i.e., time span in hours or days) were the majority (*N* = 47; 62.66%). Most of them (*N* = 25) were the laboratory experiments. Long-term studies (*N* = 28; 37.34%) included studies of time span in weeks (*N* = 11), months (*N* = 16), and years (*N* = 1). In long-term studies, most of them belong to qualitative study in field setting (*N* = 15) and field experiment (*N* = 8).

#### Summary for RQ2

In response to RQ2, the findings regarding evaluation methods are summarized as follows.

Among four types of system evaluation methods, the most common was laboratory experiment for evaluating user system interaction and system validation. In most situations, researchers evaluated target users about their behaviors, attitudes and intentions. However, proxy users (e.g., blindfolded participants, young proxies, non-disabled participants) were still adopted in evaluations, especially for testing system function in an initial stage. Some 42.47% evaluations were conducted in field settings. The typical locations were the participants’ own home and institutions. Field settings were often used for older adults and children, which could indicate an age-related issue. In qualitative studies, researchers created ad-hoc settings or in-lab settings to qualitatively evaluate the developed systems. Among all system evaluation studies, only five studies clearly reported they received an ethic approval from relevant authority (e.g., university ethical committee). The rest of the studies primarily used the consent forms for the participants.

Short-term evaluations were the majority (62.66%), primarily including (1) laboratory experiment, (2) qualitative study in artificial setting, and (3) qualitative study in field setting. Long-term evaluations (37.34%) mostly consisted of qualitative study in field setting and field experiment.

## Discussion

Based on an analysis of 65 papers related to system design and evaluation, we present insights from the following aspects: (1) HCI technologies, (2) user/stakeholder involvement in the design phase, (3) involving proxy users in laboratory experiments, and (4) engaging field setting in system evaluation. Finally, due to the impact of the coronavirus pandemic, we also identify a promising domain of promoting the online social experience.

### Insights for HCI technologies

The studies reviewed in this paper have presented a wide range of systems designed to socially assist users. Various HCI technologies have been developed and applied in these systems to serve different social purposes. In this review, we summarized 11 types of technologies, and they are often adopted in combinations to construct systems.

*Cognition and meaning understanding technologies* are often applied with wearable technologies to directly compensate for users’ sensory loss in their daily lives ([42, 44, 45, 47, 57]). Our results show that all of the reviewed studies applied such technologies to detect visual cues, which uncovers the need to design systems for other social signals.

*Wearable technologies* are useful to follow users’ daily activities and continuously provide social support. However, wearing such devices is often associated with a higher risk of privacy violations and stigmatization. Our results show that none of the studies specially considered privacy issues, and only [64] specialized on dignify users. Hence, more considerations in these aspects should be encouraged when designing related systems.

*Social network and communication technologies* are useful to assist people with mobility issues because they are more likely to feel isolated. They can be applied with the combinations with multimedia technologies ([66, 67]) to promote content sharing, human interface technologies [76] to improve accessibility, or VR [71] to create immersive social environments. Besides, although rarely investigated in our reviewed papers, [67] revealed a promising domain to enhance the online social experience for those who have difficulties in face-to-face communications.

*Sensor and device network technologies* Apart from the systems that mainly addressed individual users, sensor and device network technologies can realize bi-directional interactions between multiple people with certain devices, which is suitable to be applied in institutions or communities. They can collaborate with wearable technologies, electronic measurement technologies or Ambient Intelligence (AmI) technologies to assist daily interaction, enhance social awareness and mutual bonding.

*Emerging technologies* The studies reviewed indicate that some emerging technologies such as AmI, VR, AR were explored much less than most of other technologies, but they showed great potential and worth digging deeper. For example, AmI was applied in only four studies so far, and all of them were designed for older adults through visual feedbacks. We believe that AmI can also be applied for other user groups with limited mobilities and more forms of feedback can be developed. VR technologies are also only adopted in four studies, and all of them used VR for therapeutic interventions, which means more diverse forms of VR applications can be explored for other purposes. Although AR technology was applied in only two studies, it was proved to be effective in training the social skills of children with ASD and providing useful social information for people with visual impairments. We believe that more AR applications can be developed to overcome various social barriers, and the potential of using AR to assist other user groups (e.g., older adults and deaf people) can also be explored.

### Insights for user/stakeholder involvement in the design phase

As found in our analysis, only less than half of the reviewed studies reported structured user or stakeholder involvement in their system design phase. However, a clear tendency could also be observed that increasingly, studies start to emphasize the importance of closely involving users in the design and development phase (rather than only in validation or deployment phases). More concretely, based on our observation, we summarize a set of potential trends that the emerging research in Socially Assistive Systems are developing toward, in terms of user/stakeholder involvement in design.

First, the roles of the involved end-users and stakeholders are changing from simply “context providers” toward more of “co-designers”, as their involving approach is changing from a traditional “user-centered design (UCD)” perspective toward a “participatory design (PD)” perspective. This perhaps echoes the earlier discussion in HCI encompassing the nuanced differences between UCD and PD ([103, 104]). In short, while PD is a kind of UCD, it aims to evolve the landscape of design with more emphases on the empowerment of the users (and stakeholders) and the humility of designers. The benefits of carefully supporting users and stakeholders’ roles as co-designers is twofold. First, it enables the users (or stakeholders) to not only tell what they feel/do in the past, but also actively shape what they desire in the future. Second, it allows the design team to gain deeper empathy and knowledge concerning the target end-users. That is because users and stakeholders are the experts of their own lived experiences, and interacting with them closely as collaborators (rather than subjects) helps designers to establish a better understanding about their tacit needs and latent desires [105]. We argue that these two benefits are especially meaningful to Socially Assistive Systems, which fundamentally aim to empower the users with dignity.

The second trend observed by us, concerns the objectives of user/stakeholder involvement, which have been changing from solely need-finding around utility and efficiency, more toward meaning making in regard to sociocultural aspects. For example, in [64], the researchers set out to support people with hearing loss beyond functional needs. Instead, they explored the aesthetic and socio-cultural needs (e.g., self-expression) of this group, by designing the form and interaction of smart jewels. More examples can be found. For instance, more and more recent studies have speculated how “others” would look at the end-users using the system (social perception), rather than only how the system functions. And this is indeed an important dimension of sociocultural needs that can considerably determine the adoption of Socially Assistive Systems.

Last but not the least, we observe a trend of user/stakeholder involvement being conducted in a more ecological way. By this we mean the user involvement activities tend to be carried out more systematically (e.g., in multiple stages of design), with heavier entanglement with users’ real-world (material and cultural) context. For instance, ethnographic methods [55] or observational techniques ([68, 92]) were carried out in the context, to make the user involvement more structured and systematic, which could help yield more authentic and ecological understandings than utilizing workshops or interviews solely.

### Involving proxy users in laboratory experiments

Most studies tested representative users and reported their feelings and perceptions towards Socially Assistive Systems. Generally, this is an important rule for design researchers and practitioners to recruit representative users in system evaluation [106]. However, sometimes it becomes problematic for investigating assistive systems that support social interactions. Example difficulties include: (1) not easy to get touch with such participants, even contact with institutions; (2) difficulty to find sufficient number of the participants to run a rigorous experimental studies; (3) difficulty to get verbal feedback from such participants (e.g., people with dementia or ASD). Due to these reasons, proxy users, or so called alternative users are still used in system evaluation. There are two primary types of proxy users. The first type refers to able-bodied people who simulate a given disability in certain situation [106]. The most common is testing blindfolded participants as a replacement of real blind people (e.g., [14, 42, 49]). In [14], researchers evaluated a prototype namely Social Glasses by recruiting both blind and blindfolded participants in laboratory experiments. They used this way to achieve a large N and high statistical power in quantitative analysis. Generally, proxy users are used in the preliminary experiments ([46, 47, 83, 85, 93, 99]). Such studies reported work in progress of the systems. Proxy users could participate in these preliminary evaluations to examine the system feasibility. The second type of proxy users indicates stakeholders who are most familiar with target users, such as family members, caregivers, experts. The typical example is internet proxy user. Older adults’ grand children could be treated as proxy users to do online shopping on behave of older adults [107]. In our reviewed papers, in [98], caregivers were treated as proxy users. Researchers examined caregivers rather than older adults themselves regarding how Socially Assistive Systems could support older adults to establish social connectedness.

### Engaging field setting in system evaluation

Although the laboratory experiments are still the mainstream in system evaluation, we found a considerable number of studies conducted in field settings, which were primarily targeted at older adults ([21, 22, 61, 66, 69, 74–76, 78, 86, 91, 97]) and children ([13, 51, 52, 55, 62, 77, 89, 92]). This might be explained by the age-related issue. The special cognitive stage of older adults and children could bring challenges for researchers to make them follow the experimental procedures, so conducting field experiments or qualitative studies in the field settings became a more acceptable form for older adults and children. Numerous studies have proved that older adults and children are vulnerable to their surrounding changes, especially for those with dementia or ASD ([108, 109]). In the field settings, the participants are more likely to perform naturally in their familiar environments, which can ensure the validity of such research by reducing “the Hawthorne Effect” [110]. Apart from the age-related issues, field setting provides an appropriate way to investigate the effect of Socially Assistive Systems on the participants’ social relationships and feelings, as well as daily social interaction, which needs to be evaluated in relatively long term. The majority of field experiments (6/11) were conducted in the participants’ own home. Different from field experiments, most qualitative studies (13/20) in the field settings were conducted in institutions, such as care centers and schools. This is partly because these locations are common social environments. Another important reason is the accessibility to the participants and external support. For example, some older adults have difficulties in accepting and using new technologies, which need external supports from institutions.

### Promoting online social experience

In recent days, due to the risk of the COVID-19 pandemic, older adults and people with disabilities are more vulnerable than other people. They have to limit in-person interactions with others as much as possible, and face-to-face communication becomes quite rare. During the isolation period, anxiety, depression, poor sleep quality and physical inactivity have been reported [111]. In this context, we could identify a promising domain that enhance the online social experience for older adults and people with disabilities. For instance, using an online social exergame to attract young people and their older family adults to play together, is not only able to promote physical activity, but also to increase the fun of games and their positive emotions [112]. In this situation, social exergaming is helpful to increase bonds with family members and reduce social anxiety. Additionally, in our reviewed papers, [67] presented a multimedia story-telling severce to support people with speech and hearing impairments to interact socially when living alone.

### Limitations

This study has several limitations. Firstly, we only searched research papers in English language and it is possible that valuable findings are reported in other languages. Secondly, the research papers were limited to search from six databases (i.e., Scopus, Web of Science, ACM, Science Direct, PubMed, and IEEE Xplore). In order to increase completeness, further more databases in social science could be added, such as ASSIA and EBSCO. Thirdly, this study is limited by the search terms used and the time period of papers published, although the focus on the past 21 years could largely guarantee this systematic review covered the most recent research studies. Fourth, we discussed the general evaluation methods of Socially Assistive Systems in this review, but still lack detailed discussions regarding data gathering in system evaluation. We could analyze date gathering methods of Socially Assistive Systems evaluation in our future work [113].

## Conclusion

This paper provides an overview of Socially Assistive Systems for older adults and people with disabilities. For this systematic review, we analyzed 65 papers from two major aspects: system design and evaluation.

In system design, our results indicated 11 types of HCI technologies that supported social interactions for target users. These technologies were often adopted in combinations to construct systems. The most common was cognitive and meaning understanding technologies, often applied with wearable devices for compensating users’ sensory loss. Some systems adopted emerging technologies such as AmI, VR, AR, which revealed great potential and worth in-depth investigation. Nearly one-third of the studies involved end-users and stakeholders in the design phase. User/stakeholders involvement were observed changing from “context providers” to “co-designers”; from merely utility-needs finding to meaning making in regard to sociocultural aspects.

In system evaluation, we identified four types of evaluation methods. The majority of studies adopted the laboratory experiments to measure user system interaction and system validation. Due to the difficulty of finding and taking target users to a specific location to participate in the study, proxy users were still used in system evaluation, especially in initial experiments. Some 42.46% of all evaluations were conducted in the field settings, primarily including the participants’ own home and institutions. In these settings, the participants could feel safe, more likely to perform naturally in their familiar environments and reduce “the Hawthorne Effect”. Finally, due to the impact of the coronavirus pandemic, we identify the research opportunity of designing Socially Assistive Systems that support online social experience.

## Data Availability

Full search strategy available from the authors on request.

## Appendix

See Table

**Table 4:** Summary of paper lists and features

No	Reference	HCI technology	Social signal perception	User involvement	Evaluation method & Time span
1	Hine & Arnott, 2002; [67]	Multimedia story-telling service (web pages)	N/A	N/A	Qualitative study in artificial setting; Time span: hours
2	Miller et al. 2007; [90]	Screen-based interface	Sign language	Several deaf participants and the deaf community were involved in the process to share their feedback on the design	Qualitative study in field settings; Time span: hours
3	Vincent et al. 2007; [82]	A pocket PC with multimedia technologies that can translate sign language into oral language	Sign language	N/A	Field experiment with pre and post intervention measures; Time span: months (3)
4	Nguyen et al. 2008; [88]	Mobile devices	N/A	The participants were involved in customization during the trials	Qualitative study in field setting; Time span: weeks
5	Dadlani et al. 2010; [22]	Sensor technology for ambient display	N/A	Primary user studies: four focus groups to evaluate design concepts	Qualitative study in field setting: 1. Interviews (a laboratory simulation of a home environment); Time span: hours; 2. Case studies; Time span: weeks (2)
6	Hirano et al. 2010; [92]	Classroom technology (collaborative visual scheduling system)	N/A	The approach was highly participatory, involving all stakeholders 1. Participatory design: - Ten educators; - Three therapists; - Observations at nine special education classrooms; 2. Two focus groups (stakeholders): N = 13 and N = 8	Qualitative study in field setting; Time span: weeks (over three weeks, 97 h of observations)
7	Krishna et al. 2010; [45]	Wearable device (computer vision, haptic technologies)	Facial expressions	N/A	Laboratory experiment (usability test for system validation); Time span: hours
8	McDaniel et al. 2010; [85]	Haptic interface	Proxemics	N/A	Laboratory experiment; Time span: hours
9	Brok & Barakova, 2010; [87]	Tangible multiagent system	N/A	N/A	Laboratory experiment; Time span: hours
10	Astell et al. 2010; [81]	Multimedia touch screen system	N/A	A user-centered design approach arising from the need expressed by caregivers and people with dementia	Laboratory experiment; Time span: hours
11	Shim et al. 2010; [68]	Web application (an online poker environment)	N/A	Exploratory field work to discover how games were learned and played	Qualitative study in field setting; Time span: hours
12	Black et al. 2011; [55]	Augmentative and Alternative Communication (AAC) technology based on mobile application	N/A	User-centered design process: 1. Interviews from caregivers/stakeholders; 2. An ethnographic study over two weeks to shadow three participating children; 3. Further information (e.g., current use of AAC equipment)	Qualitative study in field setting; Time span: months (> 6)
13	Gilfeather-Crowley et al. 2011; [93]	ESA network (wearable device)	Social space, social proximity	N/A	Laboratory experiment (for system validation); Time span: hours
14	Escobedo et al. 2012; [13]	Mobile assistive application ( augmented reality)	N/A	N/A	Qualitative study in field setting; Time span: weeks (7) Pre-deployment for three weeks, deployment for three weeks and post-deployment for one week
15	Fuchsberger et al. 2012; [73]	An online platform for ambient assisted living (AAL)	N/A	Directly involved end users into different studies: 1. Workshops; 2. Interviews with end users; 3. Survey; 4. Expert interviews	Laboratory experiment; Time span: hours (1.5–2)
16	Hermann et al. 2012; [47]	Wearable device (motion sensor)	Head movements and head gestures	N/A	Laboratory experiment; Time span: hours
17	Wu & Koon, 2012; [76]	Software application with tangible devices	N/A	N/A	Qualitative study in field setting; Time span: N/A
18	Hourcade et al. 2012; [89]	Applications based on multitouch tablets	N/A	Work with 16 elementary school children and 10 middle school children over a wide range of ASD	Qualitative study in field setting; Time span: weeks
19	Garattini et al. 2012; [66]	Communication system prototype (touch screen)	N/A	Participatory design which have been reported in previous two papers	Qualitative study in field setting; Time span: months (10 weeks)
20	Magee & Betke, 2013; [23]	Embodied interface for social network	N/A	N/A	Qualitative study in field setting; Time span: hours
21	Nijhof et al. 2013; [78]	Multiple commercial devices (TV, radio, telephone and treasure box)	N/A	N/A	Qualitative study in field setting; Time span: hours
22	Anam et al. 2014; [42]	Smart glasses	Facial and behavioral expression	Participatory design, interviews and collaborate with three representative users	Laboratory experiment; Time span: hours (each conversation lasted for 10 min)
23	Bala et al. 2014; [83]	Haptic interface (a vibrotactile chair)	Facial expressions)	N/A	Laboratory experiments (usability test for system validation); Time span: hours
24	Purves et al. 2014; [80]	Touch screen based interface	N/A	Focus groups with older adults to identify and select relevant contents	Qualitative study in artificial setting; Time span: hours
25	Terven et al. 2014; [46]	Head Pose Estimation based on SIFT features and Hidden Markov Models	Gestures and postures	N/A	Laboratory experiment; Time span: hours
26	Louanne E. Boyd et al. 2015; [77]	Collaborative gaming (commercially iPad game)	N/A	N/A	Qualitative study in field setting; Time span: weeks
27	Nazzi et al. 2015; [91]	Mobile/handheld interfaces	N/A	Co-design in-situ enactments and workshops	Qualitative study in field setting; Time span: years (> 1)
28	Abdallah et al. 2016; [56]	Mobile application	Sign language	N/A	Field experiment; Time span: months (> 1)
29	Bekele et al. 2016; [96]	VR system (multimodal virtual reality-based social interaction platform)	Facial and lip expressions	N/A	Laboratory experiment (one hour); Time span: hours
30	Buimer et al. 2016; [44]	Wearable device	Facial expressions	N/A	Laboratory experiments (three phases: baseline, training, and experiment); Time span: hours (1.5)
31	Kim et al. 2016; [95]	Assistive technology (an audio-augmented badminton game)	N/A	N/A	Qualitative study in field setting (i.e., sports center); Time span: hours
32	Sauvé, et al. 2016; [69]	Online educational game	N/A	N/A	Field experiment (“pre-test/post-test” single group methodology); Time span: months (> 1)
33	Tapia et al. 2016; [79]	Smart TV applications (Google Chromecast Technology)	N/A	N/A	Laboratory experiment: Stage 1: Laboratory-based inspection (a cognitive walkthrough by authors; heuristic evaluation by expert users); Stage 2: Usability test in a lab (simulate a home setting); Time span: hours
34	Wang et al. 2016; [54]	A mobile application based on current reading assistant technologies	N/A	User centered design	Qualitative study in artificial setting; Time span: hours
35	Zhao et al. 2016; [43]	A facial landmarking technology and a facial expression recognition technology	Facial expressions	N/A	Laboratory experiment (test recognition rates) Time span: hours
36	Voss et al. 2016; [51]	Smart glasses system and mobile application	Facial expressions and head pose	N/A	Qualitative study in field setting; Time span: months (4)
37	Qiu et al. 2016; [49]	Wearable device	Gaze behaviors	N/A	Laboratory experiment; Time span: hours
38	Baez et al. 2017; [70]	Mobile application	N/A	N/A	Laboratory experiment; Time span: months (10 weeks)
39	Bonnin et al. 2017; [62]	Wearable device (smart band)	N/A	N/A	Qualitative study in field setting; Time span: weeks (continuous for up to three weeks)
40	Davis et al. 2017; [98]	Ambient assisted living (AAL)	N/A	N/A	Laboratory experiment; Time span: hours (1)
41	Gugenheimer et al. 2017; [94]	Assistive technology (collaborative technology for communication)	Vocal behavior	A focus group to gain a deeper understanding of challenges of deaf people’s experience with assistive technologies (two mockup prototypes as technology probes)	Laboratory experiment; Time span: hours (1.5–2)
42	Meza-de-Luna et al. 2017; [60]	Smart glasses (video camera and haptic belt)	Head nodding	N/A	Laboratory experiment; Time span: hours
43	Papa et al. 2017; [72]	TV-based interface	N/A	N/A	Qualitative study in artificial setting; Time span: hours
44	Wu et al. 2017; [58]	Computer vision recognition and artificial intelligence (automatic alt-text)	N/A	Iterative user centered design	1. Qualitative study in artificial setting; Time span: hours; 2. Qualitative study in field setting; Time span: weeks (2)
45	Feng et al. 2017; [86]	Interactive Table with tangible interfaces	N/A	N/A	Qualitative study in field setting; Time span: hours (in different days from 14:30 until 16:00)
46	Zolyomi et al. 2017; [59]	A head-mounted camera system with computer vision technology	N/A	N/A	Qualitative study in artificial setting; Time span: N/A
47	Rahman et al. 2017; [41]	A smart-phone based system	Facial behaviors and head pose	N/A	Laboratory experiment; Time span: hours
48	Sarfraz et al. 2017; [50]	Wearable device	Gaze behaviors	N/A	Qualitative study in artificial setting; Time span: hours
49	Louanne E. Boyd et al. 2018; [25]	Virtual Reality (VR)	Proximity, personal space, speech volume	Working in cooperation with a group of developmentally disabled adults: Stage 1: use card sort with 10 design partners to eliciting users requirements; Stage 2: role play with two participants to built low fidelity prototype; Stage 3: 10 developmentally disabled adults and 2 staff members participated in the development of high fidelity technology prototypes	Laboratory experiment (alternating treatment design); Time span: hours (20 one-minute trials in the system resulting in a total of 180 sessions)
50	Johnson et al. 2018; [100]	Affective computing (affective avatar system)	Facial expressions for six emotional states	N/A	Laboratory experiment; Time span: hours (Participants performed 20 interactions with the avatar)
51	Lin et al. 2018; [97]	VR technology	N/A	N/A	Field experiment (pre-test post-test); Time span: weeks (2)
52	McDaniel et al. 2018; [84]	Haptic interface	Facial expressions	N/A	Laboratory experiment; Time span: hours
53	Pingali et al. 2018; [99]	Smart wheel chair	Social /conversation distance	N/A	Laboratory experiment; Time span: hours
54	Yurkewich et al. 2018; [75]	A tablet-based communication technology	N/A	Iterative user-centered design	Qualitative study in field setting; Time span: months (12 weeks)
55	Fleury et al. 2019; [63]	Wearable assistive device (a fabric-based wearable SGD, wearable communication aid)	N/A	N/A	Qualitative study in field setting (i.e., school, playgroup, home, and other); Time span: weeks (4)
56	Isaacson et al. 2019; [74]	Advanced Communication Technologies (ACT) (Uniper-Care Technologies)	N/A	N/A	Field experiment; Time span: months (4–5 weeks)
57	Marti & Recupero, 2019; [64]	Wearable, smart jewels	N/A	The study adopts a participatory design approach, to understand the societal, cultural and usage scenarios	Qualitative study in artificial setting; Time span: days
58	McCarron et al. 2019; [57]	Smart watch	Face identification	N/A	Laboratory experiment; Time span: months (Over 6 months, measured three times: baseline, 3 months and 6 months)
59	Tamplin, et al. 2019; [71]	Online virtual reality platform	N/A	Two-phase iterative design	Laboratory experiment; Time span: hours
60	Lee et al. 2020; [53]	Wearable device	Face and head pose estimation	Online survey Participants recruited through Amazon Mechanical Turk, responded to technology attitude and other questions	Field experiment (in-situ study); Time span: hours
61	Li et al. 2020; [21]	Multimedia technology	N/A	Semi-structured interviews with older residents and caregivers were conducted in a Dutch nursing home, to understand the status of their social interaction	Qualitative study in field setting; Time span: days /months
62	Qiu et al. 2020; [14]	Wearable device	Gaze behaviors (e.g., eye contact)	N/A	Lab experiment; Time span: hours
63	Theil et al. 2020; [65]	Augmentative and Alternative Communication (AAC) device	Vocal behavior	Concept design was based on the findings of interviews conducted with 60 individuals living with deafblind people	N/A
64	Bellini et al. 2021; [61]	Wearable technology	N/A	N/A	Qualitative study in field setting; Time span: months
65	Hsieh et al. 2021; [52]	Eye-gaze assistive technology (EGAT)	Gaze behaviors	N/A	Field experiment (within-subjects design); Time span: hours

4.

## Abbreviations

AAL: Ambient assisted living;
AI: Artificial intelligence;
AmI: Ambient intelligence;
APA: American psychological association;
AR: Augmented reality;
ASD: Autism spectrum disorder;
AT: Assistive technology;
HCI: Human–computer interaction;
ICT: Information and communications technology;
IRB: Institutional review board;
MeSh: Medical subject heading;
PD: Participatory design;
SAA: Social-aware assistant;
SAR: Social assistive robot;
SAS: Socially assistive system;
UCD: User-centered design;
UCT: Uniper-care technology;
VR: Virtual reality
